# Effects of electronic screen exposure time on hypertensive disorders in pregnancy: a retrospective cohort study

**DOI:** 10.1186/s12889-024-18793-3

**Published:** 2024-05-21

**Authors:** Shaidi Tang, Yun Liu, Ying Gu, Qianqian Yang, Qian Wang

**Affiliations:** 1 Department of Education and Science, Liyang People’s Hospital, Changzhou, 213300 Jiangsu China; 2 Department of Obstetrics, The Affiliated Wuxi Maternity and Child Health Care Hospital of Nanjing Medical University, Wuxi, 214000 Jiangsu China; 3 Department of Community Health, Service Center for Maternal-Child Health Care and Birth Control of Xinwu District, Wuxi, 214028 Jiangsu China

**Keywords:** Hypertension, Maternal exposure, Pregnancy, Screen time

## Abstract

**Background:**

We previously conducted a case-control study and found that exposure to electronic screen before nocturnal sleep was associated with hypertensive disorders in pregnancy (HDP). Hence, we carried out this cohort study aiming to identify the effects of screen exposure time on the incidence rate and severity of HDP.

**Methods:**

A retrospective cohort study was conducted from January 2022 and July 2022 from three hospitals in Wuxi and Changzhou cities. A total of 732 women were recruited and the information included socio-demographic characteristics, screen exposure and outcomes. Generalized estimating equations and binary non-conditional logistic models were applied to multivariate analysis, calculating the odds ratios (*OR*s) and 95% confidence intervals (*CI*s) of screen exposure time.

**Results:**

The duration order of total screen time was smartphone > computer > television, while the duration order of screen time before nocturnal sleep was smartphone > television > computer. Multivariate analyses showed that the susceptibility of HDP among women who exposed to television before nocturnal sleep was 81.5% percent higher than those not exposed (*P* = 0.018, *OR*[95%*CI*] = 1.815[1.106–2.981]). In addition, total daily exposure time of television in the third trimester of pregnancy significantly increased the severity of HDP (*P* = 0.021, *OR*[95%*CI*] = 3.641[1.213–10.927]).

**Conclusions:**

Based on this preliminary study, we would suggest that pregnant women do not watch television before nocturnal sleep. While in the third trimester of pregnancy, total exposure time of television should be limited. Investigations from other areas and experimental studies should be conducted to verify the conclusion.

**Supplementary Information:**

The online version contains supplementary material available at 10.1186/s12889-024-18793-3.

## Background

Nowadays, digital devices with electronic screens have become important and widely available tools in our daily lives. As the Internet and 4G/5G network is routinely used, digital devices (including computers, smartphones and televisions) can provide all kinds of service, such as online courses, social communication, e-shopping, games, videos and so on. However, this is usually followed by much more electronic screen exposure time, which can lead to negative health effects. For direct impacts, prolonged screen viewing time can result in dry eye [1], neck and shoulder pain [2], decrease of physical activity [3] and shortened sleep duration [4]. Gradually, indirect effects may occur, including obesity [5], high blood pressure [6], variation of lipoprotein parameters [7] and depression [8]. Notably, women were more susceptible to cardiometabolic illnesses due to screen exposure time. In adult women, it was found that VO_2max_ of frequent (≥ 3 h/d) TV viewers was significantly lower than that of both moderate (1–2 h/d) or infrequent (< 1 h/d) viewers [9]. Among middle-aged women, screen time was associated with total cholesterol and low-density lipoprotein cholesterol levels [10].

Prolonged exposure to electronic screen is also very common in pregnant women. A cross-sectional survey conducted in 2015 indicated that about 25.1% reported prolonged television viewing (≥ 2 h/d), 20.6% reported prolonged computer viewing (≥ 2 h/d), and 62.6% reported prolonged mobile phone viewing (≥ 1 h/d) [11]. A case-control study carried out in 2019 showed that the median using time of smartphone and computer among healthy pregnant women was 5.5 and 3.0 h/day, respectively [12]. Therefore, it is necessary to investigate the effect of screen exposure time on the pregnant outcomes. One of few relevant studies showed that excessive mobile phone use during pregnancy might be a risk factor for lower birth weight and infant emergency transport [13]. Similarly, maternal early pregnancy sedentary time (total daily time of watching televisions or videos and working with computers) might be related to infant underweight at 12 months [14]. Nevertheless, we have not yet found any studies focusing on the effect of screen exposure time on cardiometabolic disease among pregnant women.

Hypertensive disorders in pregnancy (HDP) is defined as: Systolic blood pressure greater than or equal to 140 mmHg and/or diastolic blood pressure greater than or equal to 90 mmHg in two occasions (1) at least 6 h apart after 20 weeks of gestation for pregnancy induced hypertension or (2) before pregnancy/before 20 weeks of gestation for chronic hypertension [15]. The four types of HDP are chronic hypertension, gestational hypertension, preeclampsia-eclampsia, and chronic hypertension with superimposed preeclampsia [16]. HDP complicates approximately 10% of pregnancies [15] and poses significant short- and long-term health burdens on women and their babies [17]. We previously conducted a case-control study and found that exposure to electronic screen before nocturnal sleep in HDP women was significantly longer than that in healthy controls [12]. Hence, we carried out this retrospective cohort study aiming to: (1) assess the screen exposure time of common electronic products during the first, second and third trimester of pregnancy; (2) identify the effects of screen exposure time on the incidence rate of HDP; and (3) identify the effects of screen exposure time on the severity of HDP.

## Methods

### Ethics approval

The present study was conducted in accordance with the Declaration of Helsinki, and was approved by the ethics committees of Liyang People’s Hospital (No. 2,021,003), the Affiliated Wuxi Maternity and Child Health Care Hospital of Nanjing Medical University (No. 2020-06-0731-08) and the Service Center for Maternal-Child Health Care and Birth Control of Xinwu District (No. EC-2,021,001). Written informed consent was obtained from all participants. For privacy purposes, we did not extract the participants’ identification card number and phone number information.

### Study design, setting and participants

This was a retrospective cohort study. Participants were recruited between January 2022 and July 2022 from three hospitals in the cities of Wuxi and Changzhou, located in economically developed Jiangsu province. The inclusion criteria of participants included (1) women aged over 18 years and capable of responding to our questionnaires; and (2) providing written informed consent. The exclusion criteria included: (1) women diagnosed with chronic hypertension, chronic hypertension with superimposed preeclampsia, diabetes mellitus, hyperthyroidism, hypothyroidism, infectious disease and serious mental illness before pregnancy; (2) women diagnosed with HDP before 20 gestation weeks; and (3) polyembryony.

### Sample size determination

We calculated the sample size by the tools provided on the website https://epitools.ausvet.com.au/?page=home. The assumptions were as follows: Considering expected incidence in unexposed 6%, assumed relative risk 2, confidence level 0.95 and power 0.8. The sample size was determined to be 708. Given the non-response rate, we enlarged the sample size and eventually recruited 732 participants.

### Questionnaire and variables

**Fig. 1:**
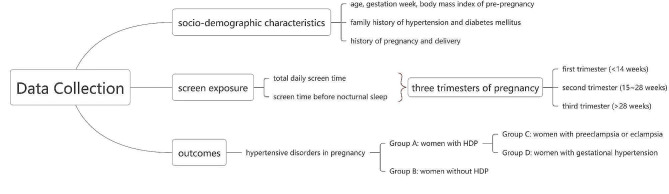
The questionnaire structure of the present study

Within 2–3 days after delivery, trained doctors might interview with participants face to face with structured questionnaires (Fig. 1). The details of questionnaires were as follows.

Socio-demographic characteristics: Information of age, gestation week, body mass index (BMI) of pre-pregnancy and history of pregnancy and delivery was extracted from the inpatient medical records. Besides, interviewers would ask about the family history of hypertension and diabetes mellitus (at least one of the parents had been diagnosed with hypertension or diabetes mellitus).

Screen exposure: In the present study, products with electronic screen included personal computers, smartphones (and/or tablet computers like iPad) and televisions. Screen time was defined as the daily average hour spent on the electronic products mentioned above, which was retrospectively self-reported by participants during the first, second and third trimesters of pregnancy. Particularly, screen time before nocturnal sleep (after 21:00 pm) was recorded.

Outcomes: The main outcome was the diagnosis of HDP, including the gestation week of onset and the type of HDP. The diagnosis guideline was adapted from 2015 recommendations of Chinese Society of Obstetrics and Gynecology [18]. It should be noted that, as one of the health care services, every pregnant woman would accept a series of antenatal examinations during the whole gestation, and all the results would be recorded in the antenatal examination books. Hence, interviewers directly extracted the diagnosis information of HDP from the books and filled in the questionnaires.

The design of questionnaire was based on the background and purpose of the present study, and then it was agreed by five obstetrician-gynecologists and two epidemiologists. Furthermore, we would ask the participants one more time if the exposure time of three trimesters of pregnancy was reported all the same. Thus, the validity and reliability could be improved.

### Grouping

All participants were distributed into two groups: Group A (women diagnosed with HDP after 20 gestation weeks) and Group B (women without HDP). There were two subgroups in Group A according to the severity of HDP: Group C (women with preeclampsia or eclampsia) and Group D (women with gestational hypertension).

### Quality control

One of the potential biases was recall bias, because the participants should recall the electronic exposure time of the first, second and third trimesters of pregnancy. We interviewed the participants face-to-face within 2–3 days of delivery, when they were in a good state, free of pain and stress. Thus, the recall bias would be reduced. The confounding bias was addressed by multivariate analyses, detailed in the next section.

### Statistical analysis

IBM SPSS Statistics 25 (IBM Corp., Armonk, NY, USA) software was used to perform statistical analysis. Since it was a retrospective cohort study and follow-up was not required, we obtained all participants’ information without any missing data. Mean ± standard deviation (*SD*) was applied to describe the continuous data if it met the normal distribution; otherwise, medians and quartiles were applied. Ratios were used to describe the enumeration data. To detect the differences between two groups, *t* test or *Mann-Whitney U* test was applied for the continuous data, depending on the normality; *χ*^*2*^ test was applied for the enumeration data. *Kruskal-Wallis H* tests and *Friedman* tests were used to examine differences in abnormal distribution variables among three groups, resting with the independence. In multivariate analysis, binary non-conditional logistic models were applied to calculate the odds ratios (*OR*s) and 95% confidence intervals (*CI*s) of screen exposure time, controlling for potential confounders. Besides, generalized estimating equations were used for repeatedly collected data. The significance level (*P*-value) was declared at 0.05.

## Results

### General characteristics

A total of 732 participants were recruited for the present study. Seventy-one women were diagnosed with HDP (Group A), and the other 661 women were not (Group B). Among Group A, there were 47 women diagnosed with preeclampsia or eclampsia (Group C), while 24 women diagnosed with gestational hypertension (Group D). Table 1 reported the general characteristics of the four groups. Except for the statistical significance of family history of hypertension between Group A and B (*χ*^*2*^=4.387, *P* = 0.036), all the other comparisons did not show any statistical differences (all *P* > 0.05). Basically, the general characteristics of the groups were balanced.

**Table 1:** Characteristics of the study participants

Variables		Categories	Group A	Group B	*P*	Group C	Group D	*P*
Age (years)	Mean ± SD^†^		30.83 ± 5.12	30.19 ± 4.35	0.250^a^	31.00 ± 4.95	30.50 ± 5.53	0.700^a^
BMI of pre-pregnancy (kg/m^2^)	n (%)	≤ 18.4	7 (9.9)	60 (9.1)	0.097^b^	5 (10.6)	2 (8.3)	0.816^b^
		18.5 ∼ 23.9	36 (50.7)	412 (62.3)		25 (53.2)	11 (45.8)	
		24.0 ∼ 27.9	17 (23.9)	138 (20.9)		11 (23.4)	6 (25.0)	
		≥ 28.0	11 (15.5)	51 (7.7)		6 (12.8)	5 (20.8)	
Family history of hypertension	n (%)	Yes	26 (36.6)	166 (25.1)	0.036^b^	18 (38.3)	8 (33.3)	0.681^b^
		No	45 (63.4)	495 (74.9)		29 (61.7)	16 (66.7)	
Family history diabetes mellitus	n (%)	Yes	59 (83.1)	592 (89.6)	0.099^c^	39 (83.0)	20 (83.3)	1.000^c^
		No	12 (16.9)	69 (10.4)		8 (17.0)	4 (16.7)	
Number of full term infants	Median (25-75%)		0 (0,1)	0 (0,1)	0.246^d^	0 (0,1)	0 (0,0)	0.187^d^
Number of premature infants	Median (25-75%)		0 (0,0)	0 (0,0)	0.301^d^	0 (0,0)	0 (0,0)	0.475^d^
Number of abortions	Median (25-75%)		0 (0,1)	0 (0,1)	0.554^d^	0 (0,1)	0 (0,1)	0.721^d^
Number of living children	Median (25-75%)		0 (0,1)	0 (0,1)	0.245^d^	0 (0,1)	0 (0,0)	0.187^d^

Group A- HDP; Group B- without HDP; Group C- preeclampsia or eclampsia; Group D- gestational hypertension

^†^ SD, standard deviation; ^‡^ BMI, body mass index

^a^*t* test; ^b^*χ*^*2*^ test; ^c^ Fisher’s exact test; ^d^*Mann-Whitney U* test

### Electronic screen exposure time during three trimesters of pregnancy

As shown in Table 2, the duration order of total screen time was smartphone > computer > television, while the duration order of screen time before nocturnal sleep was smartphone > television > computer. Then we used *Friedman* tests to determine the variation tendency of the screen exposure time during three trimesters of pregnancy. There were statistical differences in the exposure time of computer and television (all *P*<0.05), but not in the exposure time of smartphone (*P* > 0.05). In other words, computer use decreased gradually throughout the pregnancy, while the opposite occurred for television viewing time.

**Table 2:** Electronic screen exposure time during three trimesters (Median [25-75%])

Exposure time	First trimester	Second trimester	Third trimester	*P* ^a^
Total screen time of computers (hours/day)	2 (0,8)	2 (0,7)	1 (0,5)	<0.001
Screen time of computers before nocturnal sleep (hours/day)	0 (0,0)	0 (0,0)	0 (0,0)	0.004
Total screen time of smartphones (hours/day)	5 (3,8)	5 (3,8)	5 (3,8)	0.884
Screen time of smartphones before nocturnal sleep (hours/day)	2 (1,2)	2 (1,2.5)	2 (1,3)	0.428
Total screen time of televisions (hours/day)	0 (0,1)	0.5 (0,2)	0.5 (0,2)	<0.001
Screen time of televisions before nocturnal sleep (hours/day)	0 (0,1)	0 (0,1)	0 (0,1)	<0.001

^a^*χ*^*2*^ test

### Association of screen exposure time with HDP

Based on the medians and quartiles of screen exposure time, we divided the data into 2 ∼ 4 categories. Firstly, we conducted univariate analyses to detect the potential association of screen exposure time with HDP. As was shown in Table 3, total daily exposure time of computer, smartphone and television, and exposure time before nocturnal sleep of computer and smartphone were not significantly related to HDP during the first and second trimesters of pregnancy (all *P*>0.05). Meanwhile, television exposure time before nocturnal sleep was significantly longer in women with HDP than those without HDP during the first (*χ*^*2*^=4.088, *P* = 0.043) and second (*χ*^*2*^=5.466, *P* = 0.019) trimesters of pregnancy.

Then, we selected generalized estimating equations to perform multivariate analyses. Controlling for age, BMI of pre-pregnancy, family history of hypertension and diabetes mellitus, television exposure time before nocturnal sleep was significantly associated with HDP (*P* = 0.018, *OR*[95%*CI*] = 1.815[1.106–2.981]). Since the total incidence rate of HDP was less than 10%, the risk ratio (*RR*) value was approximately equal to *OR* value. In other words, the susceptibility of HDP among women who were exposed to television before nocturnal sleep was 81.5% percent higher than those not exposed.

**Table 3:** Differences of screen exposure time between women with and without HDP.

Screen exposure time (hours/day)		First trimester (n [%])			Second trimester (n [%])		
		Group A	Group B	*P* ^a^	Group A	Group B	*P* ^a^
Total screen time of computers	≤ 1.9	39 (54.9)	293 (44.3)	0.176	37 (52.1)	313 (47.4)	0.200
	2.0 ∼ 4.9	11 (15.5)	98 (14.8)		15 (21.1)	98 (14.8)	
	5.0 ∼ 6.9	8 (11.3)	68 (10.3)		7 (9.9)	69 (10.4)	
	≥ 7.0	13 (18.3)	202 (30.6)		12 (16.9)	181 (27.4)	
Screen time of computers before nocturnal sleep	0	56 (78.9)	546 (82.6)	0.435	56 (78.9)	550 (83.2)	0.358
	≥ 0	15 (21.1)	115 (17.4)		15 (21.1)	111 (16.8)	
Total screen time of smartphones	≤ 2.9	13 (18.3)	108 (16.3)	0.859	9 (12.7)	102 (15.4)	0.820
	3.0 ∼ 4.9	17 (23.9)	173 (26.2)		21 (29.6)	172 (26.0)	
	5.0 ∼ 7.9	20 (28.2)	207 (31.3)		25 (35.2)	219 (33.1)	
	≥ 8.0	21 (29.6)	173 (26.2)		16 (22.5)	168 (25.4)	
Screen time of smartphones before nocturnal sleep	≤ 0.9	11 (15.5)	75 (11.3)	0.319	10 (14.1)	72 (10.9)	0.384
	1.0 ∼ 1.9	24 (33.8)	192 (29.0)		25 (35.2)	200 (30.3)	
	2.0 ∼ 2.9	25 (35.2)	234 (35.4)		24 (33.8)	220 (33.3)	
	≥ 3.0	11 (15.5)	160 (24.2)		12 (16.9)	169 (25.6)	
Total screen time of televisions	≤ 0.9	34 (47.9)	375 (56.7)	0.158	31 (41.7)	349 (52.8)	0.185
	1.0 ∼ 1.9	14 (19.7)	138 (20.9)		15 (21.1)	144 (21.8)	
	≥ 2.0	23 (32.4)	148 (22.4)		25 (35.2)	168 (25.4)	
Screen time of televisions before nocturnal sleep	0	37 (52.1)	425 (64.3)	0.043	33 (46.5)	402 (60.8)	0.019
	> 0	34 (47.9)	236 (35.7)		38 (53.5)	259 (39.2)	

Group A- HDP; Group B- without HDP

^a^*χ*^*2*^ test

### Association of screen exposure time with the severity of HDP

Based on the medians of screen exposure time, we divided the data into 2 categories. In univariate analyses (Table 4), total daily exposure time of computer and smartphone, and exposure time before nocturnal sleep of computer, smartphone and television were not significantly related to the severity of HDP during the three trimesters of pregnancy (all *P*>0.05). However, total daily exposure time of television was significantly longer in Group C than that of Group D during the third trimester of pregnancy (*χ*^*2*^=4.589, *P* = 0.032), whereas the first and second trimesters were not (all *P*>0.05).

We also performed multivariate analyses by logistic models. Controlling for age, body mass index (BMI) of pre-pregnancy and family history of hypertension, total daily exposure time of television in the third trimester of pregnancy was significantly associated with the severity of HDP (*P* = 0.021, *OR*[95%*CI*] = 3.641[1.213–10.927]).

**Table 4:** Differences of screen exposure time between Group C and D

Screen exposure time (hours/day)		First trimester (n [%])			Second trimester (n [%])			Third trimester (n [%])		
		Group C	Group D	*P* ^a^	Group C	Group D	*P* ^a^	Group C	Group D	*P* ^a^
Total screen time of computers	≤ 4.9	32 (68.1)	18 (75.0)	0.546	34 (72.3)	18 (75.0)	0.811	35 (74.5)	20 (83.3)	0.398
	≥ 5.0	15 (31.9)	6 (25.0)		13 (27.7)	6 (25.0)		12 (25.5)	4 (16.7)	
Screen time of computers before nocturnal sleep	0	39 (83.0)	17 (70.8)	0.236	39 (83.0)	17 (70.8)	0.236	40 (85.1)	17 (70.8)	0.153
	≥ 0	8 (17.0)	7 (29.2)		8 (17.0)	7 (29.2)		7 (14.9)	7 (29.2)	
Total screen time of smartphones	≤ 4.9	19 (40.4)	11 (45.8)	0.663	19 (40.4)	11 (45.8)	0.663	18 (38.3)	13 (54.2)	0.202
	≥ 5.0	28 (59.6)	13 (54.2)		28 (59.6)	13 (54.2)		29 (61.7)	11 (45.8)	
Screen time of smartphones before nocturnal sleep	≤ 1.9	22 (46.8)	13 (54.2)	0.557	23 (48.9)	12 (50.0)	0.932	24 (51.1)	15 (62.5)	0.360
	≥ 2.0	25 (53.2)	11 (45.8)		24 (51.1)	12 (50.0)		23 (48.9)	9 (37.5)	
Total screen time of televisions	≤ 0.9	19 (40.4)	15 (62.5)	0.078	17 (36.2)	14 (58.3)	0.075	15 (31.9)	14 (58.3)	0.032
	≥ 1.0	28 (59.6)	9 (37.5)		30 (63.8)	10 (41.7)		32 (68.1)	10 (41.7)	
Screen time of televisions before nocturnal sleep	0	23 (48.9)	14 (58.3)	0.453	20 (42.6)	13 (54.2)	0.353	19 (40.4)	14 (58.3)	0.152
	> 0	24 (51.1)	10 (41.7)		27 (57.4)	11 (45.8)		28 (59.6)	10 (41.7)	

Group C- preeclampsia or eclampsia; Group D- gestational hypertension

^a^*χ*^*2*^ test

## Discussion

We conducted this retrospective cohort study from January to July in 2022, recruiting a total of 732 participants and recording their screen exposure time. We found that (1) pregnant women had spent the longest time on the smartphone among the three types of electronic products; (2) television exposure time before nocturnal sleep was significantly associated with HDP; (3) total daily exposure time of television in the third trimester of pregnancy was significantly related to the severity of HDP; (4) the computer screen exposure might not be a risk factor for HDP.

Recently, smartphones have rapidly become important and widely available tools that are routinely used for a variety of purposes by different individuals, including pregnant women. Lu found that Japanese women during pregnancy tended to excessively use mobile phones [13]. Among normal users, the average mobile using time was about 2 h/d, while the average using time of excessive users was about 5 h/d [13]. In Mexican adults, the mean (SD) hours per day of screen-based sedentary time in men was 3.6 (0.4) and in women was 2.8 (0.2) [19]. A similar situation prevailed in China. The median using time of smartphone among healthy pregnant women was 5.5 h/d according to our previous study [12], while the present study reported 5 h/d. We had thought the total daily exposure time of smartphone might increase the incidence rate of HDP, but we didn’t find the statistical significance. The reason might be the too widely use of smartphone among the participants of this study. Nevertheless, we strongly advise the pregnant women against prolonged exposure to smartphone, since it is usually followed by other negative health effects. For example, excessive mobile phone use during pregnancy might be a risk factor for lower weight at birth [13] and 12 months [14]. Moreover, in our another unpublished paper, the total daily smartphone viewing time was significantly associated with depression among women in early pregnancy.

A meta-analysis showed that high levels of total sedentary behaviour and television viewing were associated with overweight/obesity, type 2 diabetes and hypertension [20]. However, the consequences of television exposure at night were even worse. In this study, television exposure time before nocturnal sleep was significantly associated with HDP. We had thought that prolonged exposure time of television at night might reduce the sleep duration and then lead to high blood pressure [21]. However, there was no statistical significance of sleep duration between Group A and B (data not shown). As the pathophysiologic mechanisms of HDP remain elusive and few studies have focused on the relationship between screen time and HDP, the reason why television exposure time before nocturnal sleep associated with HDP was ambiguous. Hence, we plan to learn the potential mechanisms including spirit, nerve, endocrine and immunity system in future studies.

Preeclampsia is defined as new onset hypertension diagnosed after 20 weeks gestation with SBP > 140 mm Hg or DBP > 90 mm Hg with proteinuria or end-organ damage [16]. Eclampsia is defined as a generalized tonic-clonic, focal, or multifocal seizure in a preeclamptic patient without another underlying cause [16]. Thus, both preeclampsia and eclampsia are more severe than gestational hypertension. There were a number of risk factors associated with preeclampsia or eclampsia, such as age ≥ 35, rural residential area, null parity, positive history of abortion, twin pregnancy, less fruit consumption, pre-pregnancy overweight, positive family history of hypertension, pre-existing hypertension, diabetes mellitus, maternal smoking and so on [22–24]. In the present study, we suggested that (1) total daily exposure time of television in the third trimester of pregnancy was significantly related to preeclampsia and eclampsia; (2) the total exposure time of television should be limited. Although we came up with a better understanding of HDP and its prevention, a long series of research would need to be done to demonstrate causality and determine policy based on it.

For the computer screen exposure, we didn’t find any statistical association with HDP in the present study. Since a variety of computer functions can be replaced by smartphones, people tend to use smartphones more frequently. As a result, computers were used mainly for work by most women [12]. Then, the exposure time of computer was shorter than that of smartphone, especially before nocturnal sleep. So, the computer screen exposure might not be a risk factor for HDP.

Finally, there were limitations in the present study. The most significant constraint was the potential for measurement error and information bias. Since the electronic screen time was reported by the participants themselves, the resulting data might not have reached the desired level of accuracy. Although we categorized self-reported screen time into 2 to 4 levels, this was insufficient to provide conclusive recommendations. Rather, it served primarily as an indicator of general trends. As more and more smartphones can provide objective data on daily screen time, we intend to forgo self-report data in future research. Then, there was also the possibility of reverse causation. Women who were having complicated pregnancies might be lower-energy and spend more time resting in bed, thereby increasing their screen time on electronic devices. In this case, the correlation strength would be amplified. In other words, the actual *OR*s might not be as elevated as indicated in the study. Lastly, our results were from two cities located in south Jiangsu province, which is one of the developed regions in China, and thus might not be representative of other districts.

## Conclusions

Based on this preliminary study, we would suggest that pregnant women do not watch television before nocturnal sleep, since it might increase the susceptibility of HDP. While in the third trimester of pregnancy, total exposure time of television should be limited within 1 h/d, otherwise it might aggravate the disease. Investigations from other areas and experimental studies should be conducted to verify the conclusions.

### Electronic supplementary material

Below is the link to the electronic supplementary material.

Supplementary Material 1

## Data Availability

All data generated or analysed during this study are included in this published article and Additional file 1.

## Abbreviations

HDP: Hypertensive disorders in pregnancy
SD: Standard deviation
OR: Odds ratio
CI: Confidence interval
BMI: Body mass index
RR: Risk ratio
